# Informal Dementia Caregivers: Current Technology Use and Acceptance of Technology in Care

**DOI:** 10.3390/ijerph18063167

**Published:** 2021-03-19

**Authors:** Daniel Wójcik, Katarzyna Szczechowiak, Patrycja Konopka, Mateusz Owczarek, Agata Kuzia, Izabela Rydlewska-Liszkowska, Małgorzata Pikala

**Affiliations:** 1 Department of Management and Logistics in Health Care, Medical University of Lodz, Poland InterDoktorMen Medical University of Lodz, 90-419 Lodz, Poland; izabela.rydlewska-liszkowska@umed.lodz.pl; 2 Wroclaw’s Alzheimer Center, 53-659 Wroclaw, Poland; szczechowiak@gmail.com; 3 Department of Epidemiology and Biostatistics, Social and Preventive Medicine of the Medical University of Lodz, 90-752 Lodz, Poland; Malgorzata.pikala@umed.lodz.pl; 4 Institute of Psychology, Polish Academy of Sciences, 00-378 Warsaw, Poland; patrycja.konopka@sd.psych.pan.pl; 5 Institute of Psychology, University of Wroclaw, 50-527 Wroclaw, Poland; mateusz.owczarek97@onet.pl (M.O.); kuziaagata@gmail.com (A.K.)

**Keywords:** socio-demographic characteristics, informal caregivers, dementia, Alzheimer’s disease, technology

## Abstract

(1) *Background*: Given the increased social isolation caused by the COVID-19 pandemic, the challenges faced by informal dementia caregivers have increased. An increasing use of technology, both in care and dementia clinical trials, depends upon caregivers’ abilities as a user. Accordingly, the aim of our study was to verify the current technology (smartphone and computer) use and acceptance in care, regarding socio-demographic variables; (2) *Methods*: Questionnaires were distributed to 102 dementia caregivers, mostly of patients with moderate dementia; (3) *Results*: The majority of participants were women (63%), and large number of them used technological devices such as a smartphone (91%) or computer (81%). Results revealed differences between age, gender, and education level on technology acceptance. Interestingly, smartphone use and acceptance seemed to be feasible, regardless of age, whereas computer use was negatively correlated with age. Technology was perceived by respondents as most useful for patients’ activities including locomotion, toileting, and meals; (4) *Conclusions*: The future of technology use in dementia care should indicate solutions tailored to individual characteristics such as new technology solutions (GPS trackers, smartphone apps, dietary intervention, and meal planning apps).

## 1. Introduction

According to the 2016 World Alzheimer Report, the number of people with dementia worldwide (46.8 million) will almost double every 20 years in our aging society, and it is expected to rise to 131.5 million by 2050 [1]. The most common cause of dementia is Alzheimer’s disease (AD), comprising 60 to 70 percent of all dementia cases [2]. Due to the Alzheimer’s Association’s 2019 report, AD was responsible for 121,404 deaths in 2017 in the United States alone, making AD the sixth leading cause of death [3]. It is also noteworthy that the total estimated worldwide cost of dementia was predicted to rise to a trillion-dollar disease by 2018 [4]; more importantly, in 2018, over 16 million informal caregivers in the US, such as spouses, children, and other family members, provided an estimated 18.5 billion hours of voluntary care to people with dementia, valued at nearly $234 billion [3]. This shows the scale of the dementia care problem prior to the COVID-19 pandemic. There is also no doubt that during the pandemic, the elderly, including dementia patients and their elderly caregivers, are especially vulnerable, raising great concerns for mental well-being and physical health outcomes in older adults [5]. Recent studies show that both the viral infection and the enforced prolonged conditions of social isolation in order to limit the spreading of the disease have a wide-ranging impact on the elderly’s mental health [6].

Dementia symptoms generally worsen over time, and the patient becomes incapable of performing daily life activities [7]. Losing the ability to live independently without the assistance of others, usually relatives, the dementia patient may present different behavioral symptoms that can strongly affect the caregiver’s life. Therefore, the dementia caregiver’s quality of life often deteriorates over the course of the disease. Accordingly, the common potential adverse consequence of caregiving is depression [7] caused by isolation, loneliness, and exhaustion of care providers [3,8]. Hence, recent findings from a meta-analysis of 17 studies revealed that the aggregate prevalence of depression among AD caregivers was 33.9% and aggregate prevalence of anxiety disorders was 43.6% [9]. Moreover, the level of stress experienced by informal caregivers seems to be the strongest predictor of a patient’s admission into a nursing home [8]. At least 60% of dementia caregivers in the US are female relatives (wives, daughters, daughters-in-law, granddaughters) [10]; the typical characteristic of informal dementia caregivers is a middle-aged or older female child or spouse of the patient [11].

Nowadays, there is a pressing demand to provide adequate support and resources in order to improve the well-being of informal caregivers and make the dementia care challenges less severe. Devices such as smartphones, tablets, and computers can be a helpful tool in alleviating the caregiver’s psychological burden, encouraging social engagement, and easing the burden of daily activities [12]. Shreve et al. reported that smartphone technology interventions could address several needs of dementia caregivers such as reducing the psychological burden and social isolation inherent in caregiving, providing access to information and resources, and helping to ensure patient’s safety and track the progression of the disease [13]. There are many forms of internet-based activities that can meet the caregiver’s needs such as web-based training, psychological and educational programs, mobile text messaging, video-recording, and chat forums [14]. Furthermore, dementia technology based on the internet (e.g., smartphone apps, wearables, computer programs, e-learning, and online platforms) can support care and improve contact with professional care personnel, enhancing the monitoring of disease progression, identifying emerging problems, and delivering professional interventions [15,16], which could be a cost-effective solution in the long-term. In a pilot study, Boessen et al. identified the family caregivers’ requirements and determined the need for reliable information regarding the disease and contact with fellow family members to be crucial [17].

Furthermore, dementia caregivers’ ease and level of usage in the digital technology field has become increasingly important in clinical trials of new AD medications. Technology seems to be a key component in recruitment, finding the adequate clinical trial, and engaging dementia caregivers as the study informants in drug development and other treatment protocols. Technology and internet use can be also helpful in encouraging caregivers to enroll into trials earlier due to their knowledge about the patient’s early symptoms of AD. Moreover, some AD clinical trials, including medication trials, emphasize the importance of active data collection using wearable and home-based sensors and apps, online assessment tools and platforms, electronic medical records, and online technologies, which, due to the disease characteristics, involve caregivers. The caregiver’s technology use and acceptance could be crucial factors in home-testing various aspects of patient’s executive functions, memory and cognition, such as online memory tests, and mobile applications for health tracking, currently use in AD clinical trials. The examples of such tools are Novel Assessment of Nutrition and Ageing (NANA) and Cambridge Neuropsychological Test Automated Battery (CANTAB)—they both have the potential to be self-administered with the support of a caregiver [15,18]. Therefore, the use of digital technology may be crucial for monitoring the disease progression, collecting data in response to drug interventions, and finally reducing costs of the trial [15,18]. However, recent data suggest that access to the new technologies is not enough to resolve dementia caregivers’ problems and ensure effective digital inclusion [19]. Therefore, improving the accessibility of the computational solutions seems to be an important factor in its effective use in dementia care. Furthermore, the reasons for technology rejection should also gain adequate attention.

Zwingmann et al. suggested that the social isolation felt by caregivers could be reduced if support groups were established in more flexible and private settings (e.g., telephone- and internet-based, small groups with individual coaching) to counteract the lack of participation due to personal and service factors (e.g., no time, service difficulties) [20]. This has become even more prevalent due to the increased social isolation experienced by caregivers during the COVID-19 pandemic.

Regarding the limited literature on technology use in the dementia care field, the present study aimed to collect the socio-demographic characteristics of informal dementia caregivers (participants) and care recipients, respondents’ current technology use, the purpose of using it, the perceived usefulness of technology in patients’ activities of daily living (ADL), and future acceptance of technology use in care. To our knowledge, it is the first study aiming to verify such a wide range of variables, hypothesized associations, and differences between groups. We hope that our findings will help developers, researchers, and IT companies to produce needed and utile apps and devices that can support informal dementia caregivers. Ultimately, our interest is to gather data which will be useful in developing solutions tailored to individual preferences.

## 2. Materials and Methods

This study was conducted to collect, identify, and verify: (1) socio-demographic characteristics of informal dementia caregivers and recipients, (2) caregivers’ current technology use regarding smartphones and computers/PCs/tablets, (3) care recipients’ dependency level based on the ADL scale, (4) informal caregivers’ dementia technology acceptance regarding the use of smartphones and computers/PCs/tablets in care.

### 2.1. Study Population and Sampling Procedures

Participant recruitment was conducted in Wroclaw Alzheimer’s Center and partner hospital’s Dementia Unit between February 2020 and January 2021. Professional personnel administered information about participation in the study to informal caregivers of all patients registered and treated at these institutions. Respondents had to meet the following inclusion criteria to participate in the study: (1) currently the primary informal caregiver (defined as a person providing voluntary care without financial compensation) of a dementia patient, (2) the ability to speak, read, and write in Polish, (3) willing and physically able to take part the study, (4) in constant contact with the dementia patient several times a week. A total of 105 informal caregivers participated in the study; however, three respondents were excluded due to missing data in the questionnaires. Therefore, the number of participants included in the study was 102.

The experiment was conducted in accordance with the ethical standards of the 1964 Helsinki declaration and its later amendments. No ethical approval was required from the institutions because this study involved only non-invasive procedures. Informed consent was collected from all respondents. All respondents were informed that they would be asked questions regarding personal information such as socio-demographical data and patient functioning. Furthermore, respondents were told that participation in the study was voluntary, questionnaires would be completed anonymously, and they could withdraw at any time for any reason. Respondents were given the choice of completing the questionnaires in person (i.e., in sit down sessions with a study psychologist) or over the phone. Due to the COVID-19 pandemic, all hygienic conditions and sanitary requirements regarding the safety of participants and study personnel were respected and carefully followed.

### 2.2. Measures

#### 2.2.1. Questionnaires

Questionnaires were performed and/or designed to collect data on (1) socio-demographic status of informal dementia caregivers and care recipients, (2) caregivers’ current technology use regarding smartphones and computers/PCs/tablets, (3) care recipients’ dependency level based on the ADL scale, (4) caregivers’ dementia technology acceptance regarding the use of smartphones and computers/PCs/tablets in care. Questionnaires took approximately 45 min to complete. We designed a short questionnaire about socio-demographic information (e.g., caregiver’s age, gender, education, caregiving relationship, and care recipient’s age) and caregiver’s current digital technology use. The questions regarding participant’s current technology use (smartphone and computer/PC/tablet) were also included.

The Katz index of independence in activities of daily living (ADL) scale was used to measure the patient’s dependency status [21]. The index ranks the patient’s level of performance in six functions: bathing, dressing, toileting, transferring, continence, and feeding, indicating the level of their functional impairment. The caregivers were asked to score yes or no for independence in each of the six domains. A score of 6 indicates full function and patient’s independence; 4 indicates moderate impairment; 2 or fewer indicates severe functional impairment [21]. We added the question “Do you think that technology could be helpful in care regarding this activity?” to each item of daily activity performed by the patient. Therefore, the first section of the questionnaire included a brief description of “technology” assisting in dementia care, defining it as “any internet-based technologies designed to assist and help in dementia care, such as a computer, tablet, smartphone, GPS tracker, robot, and electronic device”.

After insightful research and data collection, we designed a questionnaire composed of the items measuring the technology acceptance construct within Wang et al.’s Unified Theory of Acceptance and Use of Technology (UTAUT) model [22]. The first section of the questionnaire referred to the acceptance of computer/PC/tablet use in care, and the second section measured the acceptance of smartphone use in care. All items were measured on a 5-point Likert-type scale, ranging from 1 (strongly disagree) to 5 (strongly agree). We modify adequately the sentences to fit both (smartphone and computer/PC/tablet) variants and chose items created by Venkatesh et al. to measure the following: (1) behavioral intention (BI)—respondents’ intention of future technology use; (2) social influence (SI)—the impact of significant others’ opinions on the respondents’ behavior toward technology use; (3) facilitating conditions (FC)—subject’s perception of the degree to which infrastructure (technical and organizational) supports technology use; (4) effort expectancy (EE)—the level of ease related to users’ use of technology; (5) performance expectancy (PE)—the degree to which respondents believe using technology will allow users to perform specific activities effectively, which seems to be a crucial factor of BI to use the technology. Furthermore, technology acceptance studies suggest that BI is a predictor for actual technology use [23,24,25], and SI is crucial for technology user acceptance [26,27].

The questionnaires were previously tested among dementia professionals to estimate the time needed to complete all items. We divided the collected data into two categories of variables: independent and dependent. Due to limited data existing in the dementia caregiving field, the study was designed to examine the associations between the socio-demographic characteristic of caregivers and patients, patient’s ADL, perceived usefulness of technology (smartphone/computer) in care, current technology use, and technology acceptance. We also aimed to verify the purpose of technology use in dementia care reported by informal caregivers. The previous studies reveal that women are more receptive towards technology use in care than men, including the use of tracking devices in dementia patients [28,29]. We hypothesized that there will be differences between groups in (1) caregivers’ socio-demographic status (gender, age, and education) on current technology use (smartphone/computer), (2) caregivers’ socio-demographic status (gender, age, and education) on technology acceptance in dementia care domains; and associations between (1) caregivers’ socio-demographic status and current technology use (smartphone/computer), and technology acceptance (smartphone/computer) in dementia care, (2) patients’ ADL and dependency level, dementia stage, and time since the first diagnosis reported by caregivers.

#### 2.2.2. Independent Variables

Dementia caregivers’ socio-demographic information was collected to describe the respondents. The variables included the caregiver’s age, gender, education, marital status, caregiving relationship, and care recipients’ age, gender, dementia stage, dependency level, time since first diagnosis, and ADL.

#### 2.2.3. Dependent Variables

The assessment of study objectives included: (1) the current level and purpose of technology use (smartphone and computer/PC/tablet); (2) the perceived usefulness of technology in everyday assistance with patients’ ADL activities; (3) the acceptance of using technology (computer/PC/tablet and smartphone) in dementia care.

### 2.3. Data Analysis

The statistical analyses were performed using Statistica 13.1 (StatSoft, Krakow, Poland). Descriptive statistics including frequency distributions, percentages, means, standard deviations, and medians were used to describe study population characteristics and examine the current level and purpose of technology use, perceived usefulness of technology in everyday assistance with patient’s ADL activities, and the acceptance of technology use in dementia care. Furthermore, t-tests were conducted to study differences in current level and purpose, perceived usefulness, and acceptance of technology use variables between groups: (1) male and female respondents, (2) caregivers younger than 65 years old, and caregivers 65 years old and older, and (3) respondents with high school education level or less, and those with higher education. To examine the correlation between socio-demographic variables, current technology use, and acceptance of technology use in several domains, we used Spearman’s rank correlation coefficient, a nonparametric measure of rank correlation, described using a monotonic function. A significance level of 0.05 was used for the analysis.

## 3. Results

The study population consisted of 102 informal caregivers of patients with dementia. The age of respondents ranged from a minimum of 27 to a maximum of 80 years old, with a mean age of 58.5 years (standard deviation = 11.8; median = 61). The majority of caregivers were women (63%). The analysis of respondents’ marital status showed 85% declared they were married, 6% were widowed, and 8% were single/divorced. Most participants had a high school diploma (53%) or higher level of education (46%); only 1% of respondents had primary education. Spearman’s rank correlation coefficients showed a medium negative correlation between the age and education level of participants (−0.43); as the age increases, the level of education decreases (see Table 1 for full list of correlations).

**Figure 1:**
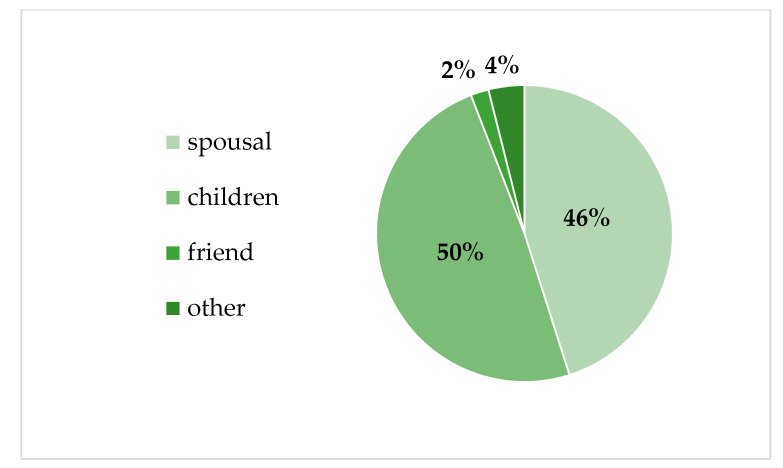
Study population characteristics—caregiving relationship.

**Figure 2:**
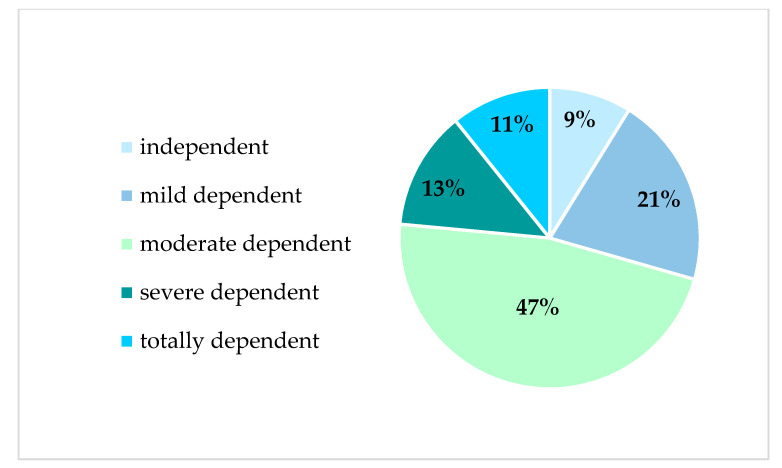
Study population characteristics—care recipient’s dependency level.

The *t*-test analyses showed no significant difference in current smartphone and computer use between caregivers 65+ and younger (Table 2) or gender groups (Table 3). Regarding the socio-demographic characteristics of dementia caregivers and care recipients, the percentage distribution of caregiving relationship (Figure 1), and care recipients’ dependency level (Figure 2) are presented below.

Care recipients’ dementia stage was categorized as early (46%), middle (44%), and late (6%) stage dementia, with the majority of care recipients being moderately dependent (47%). The mean time since initial diagnosis was 2.2 years (SD = 1.9) and ranged from less than a year to a maximum of 10 years. Furthermore, over half (56%) of dementia patients were female.

**Figure 3:**
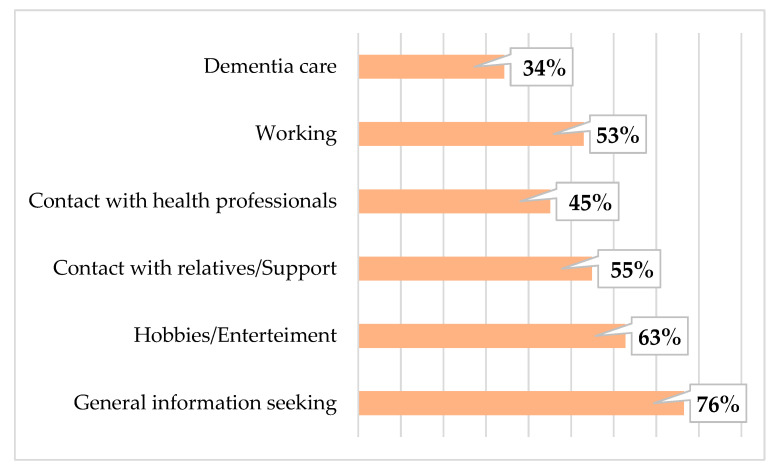
The purpose of computer use in caregivers.

**Figure 4:**
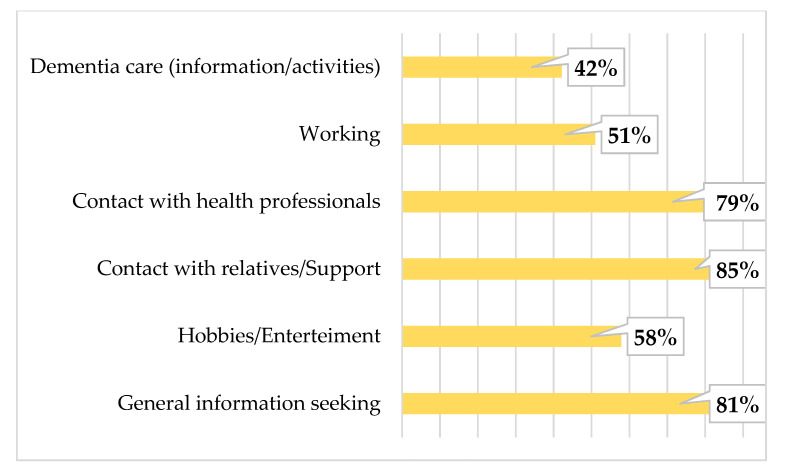
The purpose of smartphone use in caregivers.

In total, 91% of respondents reported they currently use a smartphone, and 81% currently use a computer (PC/tablet). As shown in Figure 3 and Figure 4, the majority of participants mostly use these devices to seek general information, including dementia-related information.

Spearman’s rank correlation coefficient showed no statistically significant association between age and smartphone use. However, a medium negative correlation between age and current computer use was found (−0.30), showing the older the caregivers were, the less often they use the computer. Moreover, there was also a small positive correlation between education level and current smartphone use (0.25), and a medium positive correlation between education and current computer use (0.41). Furthermore, there was a strong positive correlation between smartphone and computer use (0.56). There were no more statistically significant relationships between the socio-demographic variables.

Table 4 shows Spearman’s rank correlation coefficients between ADL score, time since initial diagnosis, dementia stage, and patient’s dependency level. There was no association between participants’ socio-demographic variables and patients’ ADL score, ADL scores in each domain, or the purpose of technology use in each activity. However, there was a strong inverse association between patients’ dependency level (in the respondents’ opinion) and ADL score (−0.51). Moreover, there were medium negative correlations between ADL score and: (1) time since first diagnosis (−0.30) and (2) dementia stage (−0.48). Accordingly, the higher the ADL score, the lower patients’ dependency level, time since initial diagnosis, and dementia stage were. On the other hand, positive correlations between patients’ dependency level and: (1) time since the first diagnosis and (2) dementia stage were observed.

**Figure 5:**
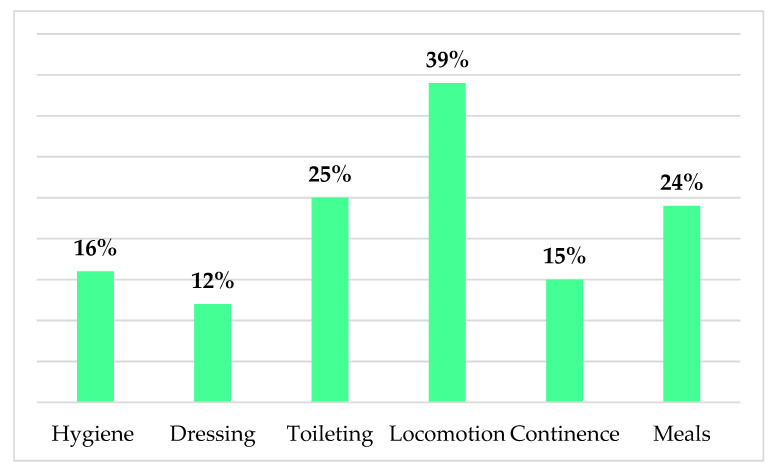
Usefulness of technology perceived by caregivers in helping with ADLs.

We also measured caregivers’ perceived usefulness of technology in helping with patients’ ADLs, illustrated in Figure 5. The majority of informal dementia caregivers perceived technology to be most useful in patients’ locomotion, toileting, and meals domains.

Acceptance of technology in dementia care was correlated with respondents’ age, education level, and current use of technology, including smartphones and computers. Hence, there were small negative correlations between respondents’ age and BI, SI, and EE related to computer acceptance in care (see Table 1). Interestingly, the strongest associations occurred as strong positive correlations between current computer use and BI (0.52), FC (0.62), and EE (0.51). There were also statistically significant differences in acceptance of computers in care between older (>65 years old) and younger (<65 years old) participants; with the younger participants scoring significantly higher in BI (*p* = 0.01), SI (*p* = 0.05), and EE (*p* = 0.02) domains (see Table 2). There were no differences between these age groups regarding smartphone acceptance. On the other hand, the significant differences between males and females included all smartphone acceptance in care domains, showing higher scores in women (see Table 3).

The differences between the group of respondents with a high school education level or less, and those with higher education, are even more interesting (see Table 5). Respondents with a higher education level used a smartphone (*p* = 0.003) and computer (*p* = 0.000003) significantly more often than those with a high school education or less. Participants with a higher education also had significantly higher scores in six of the ten acceptance domains related both to smartphone and computer use in care, than those with the level of high school education or less.

## 4. Discussion

The wide range of variables allows us to examine complex associations between measured factors and verify several hypotheses regarding technology use. Meanwhile, the mean age of respondents was 58.5 years, only 31% of them were age 65+. The majority of participants were women, and a large number. The majority of participants reported currently using technological devices such as smartphones (91%), and computers (81%). The main purposes of smartphone use in daily living were contact with relatives/support, seeking information, and contact with health professionals. These may also have been influenced by the COVID-19 pandemic, social distancing restrictions, and limited face-to-face contact. On the other hand, caregivers use the computer mainly for information seeking and hobbies/entertainment purposes. These differences between purposes in smartphone and computer use could help create new apps and programs for dementia caregivers.

The current study confirmed previous findings that the main reason dementia caregivers use a smartphone is to seek support. The literature has consistently shown that, given the stressful nature of caregiving, internet-based digital tools, online platforms, and support groups play an important role in regaining a sense of social inclusion and belonging, reported by caregivers [30] Online support systems, such as the online social support intervention reported by Dam et al. [31] and/or the videophone psychosocial intervention conducted by Czaja et al. [32] should therefore be taken into consideration, especially during the COVID-19 pandemic. Czaja et al. concluded that caregivers who received the videophone psychosocial intervention reported improvement in their caregiving skills, a decrease in burden, an increase in perceived social support, and positive perceptions of the caregiving experience [32], which included most of the difficulties experienced by caregivers.

Interestingly, since most care recipients were moderately dependent (47%), the technology was perceived by respondents as most useful in patients’: (1) locomotion, (2) toileting, and (3) meals, based on ADL domains. This highlights the fields of potential dementia care needs in creating new solutions, and electronic devices such as GPS trackers, smartphone apps, dietary intervention apps. Accordingly, recent data [33] show that care recipients’ wandering, caregivers’ concern, and caregivers’ smartphone usage can predict informal caregivers’ BI to use GPS tracking devices. In line with our findings regarding the perceived usefulness of technology in managing patients’ meals and diet, recent data show that electronic devices such as intelligent voice assistant technology in preparing meals could be a valuable solution for dementia caregivers even when they do not have a technical background [34]. The impact of this could be far reaching, as research suggests that dietary interventions, such as an anti-inflammatory diet [35] and MIND diet - Mediterranean-DASH (Dietary Approaches to Stop Hypertension) Intervention for Neurodegenerative Delay [36], can slow the progression of AD. The large body of evidence shows that, given the stressful nature of caregiving, internet-based digital tools, online platforms, and support groups play an important role in regaining a sense of social inclusion and belonging, reported by caregivers [36]. Moreover, our findings show, not surprisingly, the longer time since the first diagnosis, the higher stage of dementia, and the higher the dependency level of care recipient were, the lower ADL score was.

As we hypothesized, there were differences between socio-demographic groups in current smartphone/computer use, but only in the case of education. The respondents with a higher education level used both smartphones, and computers more frequently. There were no differences in technology use between men and women, nor between the younger and 65+ caregivers. However, there were correlations between age, education, and current technology use, indicating that the older caregivers were, the less often they use the computer. Furthermore, there was a significant association between smartphone and computer use. Finally, the higher the level of caregivers’ education, the more often they use both the smartphone and computer. Interestingly, the level of education decreases as age increases, which could be an additional factor in technology use difficulties, as mentioned in previous studies [20].

We hypothesized that technology acceptance in dementia care domains would differ depending on caregivers’ socio-demographic status (gender, age, and education). The findings confirmed differences between groups, which may be crucial in providing adequate technology solutions for informal dementia caregivers and meeting their needs and expectations. Current computer and smartphone use were both positively correlated with computer and smartphone BI—the domain often used to predict technology user actual behavior [22]. However, the role of SI, the encouraging function of significant others influencing user behavior, had no association with age, education level, gender, or current technology use. Furthermore, women and respondents with higher education were more likely to accept technology in care, which can be a crucial factor in matching new technology solutions to caregivers and their needs.

We perceive our small sample findings as an introduction to a larger, more widely measured study. Our study is not exempt from limitations. Therefore, additional socio-demographic information such as household income levels, work/occupation, urban/rural residency, financial abilities to buy adequate apps and/or devices, and type of dwelling should be taken into consideration in future studies. Moreover, the missing data of three respondents make carefully checking and cautionary data collection the priority. A qualitative analysis of caregivers’ needs regarding dementia care technology could also bring more data to the research in future perspectives.

## 5. Conclusions

Given the enormous unpredictability of the COVID-19 pandemic, social distancing, and face-to-face contact restrictions, the level of challenges faced by informal dementia caregivers increases. Therefore, the role of technology in caregiving daily activities has gained considerable significance in the past year. Due to the growing use of technology in dementia (especially AD), clinical trials (recruitment and participation conducted via technology use), helpfulness in care, and insufficiency of effective digital inclusion to improve the well-being of dementia caregivers, there is a pressing demand to identify the factors responsible for improved accessibility of technology devices. Accordingly, our study aimed to verify the level of current technology (smartphone and computer) use and future acceptance of these technology devices in dementia care, as well as socio-demographic factors of informal caregivers and care recipients.

Our findings reveal that computer use decreases with age; however, this effect of age did not smartphone use. Moreover, the higher the level of caregivers’ education, the more often they use both a smartphone and computer. Noteworthy, the level of education decreased as age increased, which could be an additional factor in technology use difficulties. An advantage of the study was measuring not only BI of smartphone and computer use in care as a predictor of future behavior towards technology, but also current use of these devices. The variables were correlated in both smartphone and computer use. Furthermore, caregivers over 65 years of age had significantly lower scores in computer use BI, SI, FC, EE, showing that age can be an important barrier to the acceptance of computer use in care. There was no such difference with the smartphone use domains, however. Therefore, interestingly, smartphone use and its acceptance in care seems to be more feasible, regardless of age. Moreover, the following differences between age, gender, and education in technology acceptance domains occurred: women were more likely to accept technology in care; there were significant differences between males and females in computer BI, and BI, SI, FC, EE, and PE; and respondents younger than 65 had significantly higher scores in BI, SI, and EE, than those older than 65 years participants.

Our findings highlight the importance of caregiver’s characteristic for technology developers. The care in the majority of cases concerned moderate dementia patients, and the technology was perceived by respondents as most useful in patients’ ADLs including locomotion, toileting, and meals. This shows the potential of developing new technology solutions such as GPS trackers, smartphone apps, dietary intervention, and meal planning apps. The types of apps seem crucial. The target population for the new technologies and devices in care are women and caregivers with higher education. Caregivers use and accept rather smartphones than computers. Therefore, smartphone apps may be more desirable in care than computer applications. The differences between purposes in smartphone and computer use should also be taken into consideration by technology companies in creating assistive technology for caregivers. While smartphone is more often used for seeking support and information, the computer seems to be more suitable for hobbies/entertainment purposes. The role of important others in technology acceptance is also an important factor in bringing technologies to market. Moreover, the importance of active data collection using wearable and home-based sensors and apps, on-line assessment tools and platforms, electronic medical records, and online technologies, which, due to the disease characteristics, involve caregivers could be beneficial and reduce costs of AD clinical trials.

In sum, the future of technology use in dementia care should indicate solutions tailored to individual characteristics and preferences. We hope that our findings will help to elucidate the specifics of caregivers as technology users.

## Figures and Tables

**Table 1:** The correlation between age, education level, current technology use, and technology use acceptance (Spearman’s rank correlation coefficient).

Variable	Spearman’s Rank Correlation Coefficient *			
	Age	Current Smartphone Use	Current Computer Use	Education Level
Age	1.00	−0.14	−0.30	−0.43
Current smartphone use	−0.14	1.00	0.56	0.25
Current computer use	−0.30	0.56	1.00	0.41
Computer_Behavioral intention (BI)	−0.25	0.35	0.52	0.49
Computer_Social influence (SI)	−0.13	0.13	0.09	0.05
Computer_Facilitating conditions (FC)	−0.23	0.42	0.62	0.30
Computer_Effort expectancy (EE)	−0.28	0.45	0.51	0.48
Computer_Performance expectancy (PE)	−0.06	0.28	0.33	0.31
Smartphone_Behavioral intention (BI)	0.10	0.42	0.32	0.23
Smartphone_Social influence (SI)	−0.02	0.19	0.00	0.10
Smartphone_Facilitating conditions (FC)	0.07	0.45	0.27	0.14
Smartphone_Effort expectancy (EE)	0.03	0.43	0.34	0.24
Smartphone_Performance expectancy (PE)	0.25	0.33	0.12	0.13

* correlation significant at the level *p* < 0.05.

**Table 2:** Difference between age groups in current technology use and technology use acceptance (T-Statistics).

Variable	T-Statistics; Grouping Variable: Age Less or Greater than 65 Years						
	Mean (<65)	Mean (>65)	t	df	*p*-Value	SD (<65)	SD (>65)
Current smartphone use	0.90	0.94	−0.61	100	0.54	0.30	0.25
Current computer use	0.83	0.78	0.56	100	0.57	0.38	0.42
Computer_Behavioral intention (BI)	11.47	9.97	2.50	100	0.01 *	2.93	2.53
Computer_Social influence (SI)	6.06	4.94	2.00	100	0.05 *	2.60	2.70
Computer_Facilitating conditions (FC)	12.67	12.09	0.87	100	0.38	3.23	2.80
Computer_Effort expectancy (EE)	15.31	13.38	2.36	100	0.02 *	3.74	4.09
Computer_Performance expectancy (PE)	13.74	12.47	1.50	100	0.14	3.91	4.10
Smartphone_Behavioral intention (BI)	11.81	12.88	−1.53	100	0.13	3.54	2.46
Smartphone_Social influence (SI)	6.34	5.63	1.22	100	0.22	2.81	2.60
Smartphone_Facilitating conditions (FC)	12.59	13.47	−1.49	100	0.14	3.11	1.83
Smartphone_Effort expectancy (EE)	15.41	15.97	−0.72	100	0.47	3.72	3.38
Smartphone_Performance expectancy (PE)	14.84	16.38	−1.82	100	0.07	4.15	3.47

* marked factors statistically significant with *p* < 0.05.

**Table 3:** Difference between gender groups in current technology use and technology use acceptance (T-Statistics).

Variable	T-Statistics; Grouping Variable: Male (M) or Female (F)						
	Mean (M)	Mean (F)	t	df	*p*-Value	SD (M)	SD (F)
Current smartphone use	0.84	0.95	−1.93	100	0.0568	0.37	0.21
Current computer use	0.76	0.84	−1.01	100	0.3169	0.43	0.37
Computer_Behavioral intention (BI)	10.13	11.52	−2.4	100	0.0183 *	2.82	2.82
Computer_Social influence (SI)	5.39	5.89	−0.91	100	0.3668	2.28	2.87
Computer_Facilitating conditions (FC)	12.13	12.7	−0.9	100	0.3703	3.45	2.88
Computer_Effort expectancy (EE)	13.79	15.25	−1.83	100	0.0698	3.48	4.11
Computer_Performance expectancy (PE)	12.61	13.78	−1.45	100	0.1514	3.67	4.14
Smartphone_Behavioral intention (BI)	10.84	12.92	−3.26	100	0.0015 *	3.19	3.07
Smartphone_Social influence (SI)	5.34	6.58	−2.23	100	0.0277 *	2.13	2.99
Smartphone_Facilitating conditions (FC)	12.11	13.31	−2.14	100	0.0344 *	3.13	2.5
Smartphone_Effort expectancy (EE)	13.92	16.58	−3.83	100	0.0002 *	4.04	2.94
Smartphone_Performance expectancy (PE)	13.76	16.25	−3.17	100	0.0020 *	3.55	3.98

* marked factors statistically significant with *p* < 0.05.

**Table 4:** The correlation between the Activities of Daily Living (ADL) score, time since the first diagnosis, dementia stage, and patients’ dependency level (Spearman’s rank correlation coefficient).

Variable	Spearman’s Rank Correlation Coefficient *			
	Time since First Diagnosis	Dementia Stage	Dependency Level	ADL Score
Time since first diagnosis	1.00	0.48	0.27	−0.30
Dementia stage	0.48	1.00	0.42	−0.48
Dependency level	0.27	0.42	1.00	−0.51
ADL score	−0.30	−0.48	−0.51	1.00

* correlation significant at the level *p* < 0.05.

**Table 5:** Difference between education level groups in current technology use and technology use acceptance (T-Statistics).

Variable	T-Statistics; Grouping Variable: High School Education or Less (HS) and Higher Education (HE)						
	Mean (HE)	Mean (HS)	t	df	*p*-Value	SD (HS)	SD (HE)
Current smartphone use	1.00	0.84	3.00	100	0.003382 *	0.00	0.37
Current computer use	1.00	0.65	4.93	100	0.000003 *	0.00	0.48
Computer_Behavioral intention (BI)	12.47	9.75	5.37	100	0.000001 *	2.35	2.72
Computer_Social influence (SI)	5.94	5.51	0.80	100	0.423128	2.78	2.58
Computer_Facilitating conditions (FC)	13.66	11.49	3.74	100	0.000303 *	2.16	3.43
Computer_Effort expectancy (EE)	16.60	13.09	4.98	100	0.000003 *	2.90	4.01
Computer_Performance expectancy (PE)	14.66	12.22	3.22	100	0.001748 *	3.94	3.72
Smartphone_Behavioral intention (BI)	12.89	11.51	2.18	100	0.031928 *	2.88	3.46
Smartphone_Social influence (SI)	6.47	5.82	1.19	100	0.236859	2.90	2.62
Smartphone_Facilitating conditions (FC)	13.43	12.38	1.90	100	0.059939	2.28	3.11
Smartphone_Effort expectancy (EE)	16.62	14.71	2.74	100	0.007178 *	3.07	3.83
Smartphone_Performance expectancy (PE)	15.98	14.76	1.54	100	0.126620	4.34	3.63

* marked factors statistically significant with *p* < 0.05.

## Data Availability

The data presented in this study are available on request from the corresponding author.

## References

[1] Prince M., Comas-Herrera A., Knapp M., Guerchet M., Karagiannidou M. (2016). World Alzheimer Report 2016: Improving Healthcare for People Living with Dementia: Coverage, Quality and Costs Now and in the Future.

[2] Mielke M.M. (2018). Sex and gender differences in Alzheimer’s disease dementia. Psychiatr. Times.

[3] Gaugler J., James B., Johnson T., Marin A., Weuve J. (2019). Alzheimer’s disease facts and figures. Alzheimers Dement.

[4] Grill J.D., Karlawish J. (2010). Addressing the challenges to successful recruitment and retention in Alzheimer’s disease clinical trials. Alzheimers Res. Ther..

[5] Wang H., Li T., Barbarino P., Gauthier S., Brodaty H., Molinuevo J.L., Weidner W. (2020). Dementia care during COVID-19. Lancet.

[6] Manca R., De Marco M., Venneri A. (2020). The Impact of COVID-19 Infection and Enforced Prolonged Social Isolation on Neuropsychiatric Symptoms in Older Adults With and Without Dementia: A Review. Front. Psychiatry.

[7] Covinsky K.E., Newcomer R., Fox P., Wood J., Sands L., Dane K., Yaffe K. (2003). Patient and caregiver characteristics associated with depression in caregivers of patients with dementia. J. Gen. Intern. Med..

[8] Alzheimer’s Association (2018). 2018 Alzheimer’s disease facts and figures. Alzheimers Dement..

[9] Sallim A.B., Sayampanathan A.A., Cuttilan A., Ho R.C.M. (2015). Prevalence of mental health disorders among caregivers of patients with Alzheimer disease. JAMDA.

[10] Alzheimer’s Association and National Alliance for Caregiving (2004). Families Care: Alzheimer’s Caregiving in the United States.

[11] Brodaty H., Donkin M. (2009). Family caregivers of people with dementia. Dialogues. Clin. Neurosci..

[12] Kay E.S., Hristidis V. (2019). Smartphone-based health technologies for dementia care: Opportunities, challenges, and current practices. J. Appl. Gerontol..

[13] Shreve J., Baier R.R., Epstein-Lubow G., Gardner R.L. (2016). Dementia caregivers’ technology preferences: Design insights from qualitative interviews. Gerontechnology.

[14] Cuffaro L., Di Lorenzo F., Bonavita S., Tedeschi G., Leocani L., Lavorgna L. (2020). Dementia care and COVID-19 pandemic: A necessary digital revolution. J. Neurol. Sci..

[15] Astell A.J., Bouranis N., Hoey J., Lindauer A., Mihailidis A., Nugent C., Robillard J.M. (2019). Technology and dementia: The future is now. Dement. Geriatr. Cogn. Disord..

[16] Klimova B., Valis M., Kuca K., Masopust J. (2019). E-learning as valuable caregivers’ support for people with dementia–A systematic review. BMC Health Serv. Res..

[17] Boessen A.B., Verwey R., Duymelinck S., van Rossum E. (2017). An online platform to support the network of caregivers of people with dementia. J. Aging Res..

[18] Gold M., Amatniek J., Carrillo M.C., Cedarbaum J.M., Hendrix J.A., Miller B.B., Czaja S.J. (2018). Digital technologies as biomarkers, clinical outcomes assessment, and recruitment tools in Alzheimer’s disease clinical trials. Alzheimers Dement..

[19] Cunha B.C., Rodrigues K.R., Pimentel M.D.G.C. Synthesizing guidelines for facilitating elderly-smartphone interaction. Proceedings of the 25th Brazillian Symposium on Multimedia and the Web.

[20] Zwingmann I., Dreier-Wolfgramm A., Esser A., Wucherer D., Thyrian J.R., Eichler T., Kilimann I. (2020). Why do family dementia caregivers reject caregiver support services? Analyzing types of rejection and associated health-impairments in a cluster-randomized controlled intervention trial. BMC Health Serv. Res..

[21] Shelkey M., Wallace M. (1999). Katz index of independence in activities of daily living. J. Gerontol. Nurs..

[22] Wang H., Tao D., Yu N., Qu X. (2020). Understanding consumer acceptance of healthcare wearable devices: An integrated model of UTAUT and TTF. Int. J. Med. Inform..

[23] Venkatesh V., Morris M.G., Davis G.B., Davis F.D. (2003). User acceptance of information technology: Toward a unified view. MIS Q..

[24] Li Q., Ma A.H.S., Chan S.S. (2019). Man, Health monitoring through wearable technologies for older adults: Smart wearables acceptance model. Appl. Ergon..

[25] Or C.K., Karsh B.T.D., Severtson J., Burke L.J., Brown R.L., Brennan P.F. (2011). Factors affecting home care patients’ acceptance of a web-based interactive self-management technology. J. Am. Med. Inform. Assoc..

[26] Gao Y., Li H., Luo Y. (2015). An empirical study of wearable technology acceptance in healthcare. Ind. Manag. Data Syst..

[27] Dwivedi Y.K., Rana N.P., Jeyaraj A., Clement M., Williams M.D. (2019). Re-examining the Unified Theory of Acceptance and Use of Technology (UTAUT): Towards a revised theoretical model. Inf. Syst. Front..

[28] Rialle V., Ollivet C., Guigui C., Herve C. (2008). What do family caregivers of Alzheimer’s disease patients desire in smart home technologies? Contrasted results of a wide survey. Methods Inf. Med..

[29] Xiong C., Astell A., Mihailidis A., Colantonio A. (2018). Needs and preferences for technology among Chinese family caregivers of persons with dementia: A pilot study. J. Rehabil. Assistive Technol. Eng..

[30] Newman K., Wang A.H., Wang A.Z.Y., Hanna D. (2019). The role of internet-based digital tools in reducing social isolation and addressing support needs among informal caregivers: A scoping review. BMC Public Health.

[31] Dam A.E., de Vugt M.E., van Boxtel M.P., Verhey F.R. (2017). Effectiveness of an online social support intervention for caregivers of people with dementia: The study protocol of a randomized controlled trial. Trials.

[32] Czaja S.J., Loewenstein D., Schulz R., Nair S.N., Perdomo D. (2013). A Videophone Psychosocial Intervention for Dementia Caregivers. Am. J. Geriatr. Psychiatry.

[33] Sun Y., Kim H.M., Xu Y., Wang Y., Kwong J., Kim S., Mclaughlin M. GPS Tracking in Dementia Caregiving: Social Norm, Perceived Usefulness, and Behavioral Intent to Use Technology. Proceedings of the 54th Hawaii International Conference on System Sciences.

[34] Li J., Maharjan B., Xie B., Tao C.A. (2020). Personalized Voice-Based Diet Assistant for Caregivers of Alzheimer Disease and Related Dementias: System Development and Validation. J. Med. Internet Res..

[35] Szczechowiak K., Diniz B.S., Leszek J. (2019). Diet and Alzheimer‘s dementia–Nutritional approach to modulate inflammation. Pharmacol. Biochem. Behav..

[36] Morris M.C., Tangney C.C., Wang Y., Sacks F.M., Barnes L.L., Bennett D.A., Aggarwal N.T. (2015). MIND diet slows cognitive decline with aging. Alzheimers Dement..

